# 2,5-Di­methyl­bufo­tenine and 2,5-di­methyl­bufo­teni­dine: novel derivatives of natural tryptamines found in *Bufo alvarius* toads

**DOI:** 10.1107/S2056989021000803

**Published:** 2021-01-29

**Authors:** Duyen N. K. Pham, Andrew R. Chadeayne, James A. Golen, David R. Manke

**Affiliations:** a University of Massachusetts Dartmouth, 285 Old Westport Road, North Dartmouth, MA 02747, USA; b CaaMTech, LLC, 58 East Sunset Way, Suite 209, Issaquah, WA 98027, USA

**Keywords:** crystal structure, tryptamines, indoles, hydrogen bonds

## Abstract

The solid-state structure of the bufotenine derivative bis­(5-meth­oxy-2,*N*,*N*-tri­methyl­tryptammonium) (5-MeO-2-Me-DMT) fumarate (systematic name: bis­{[2-(5-meth­oxy-2-methyl-1*H*-indol-3-yl)eth­yl]di­methyl­aza­nium} (2*E*)-but-2-enedioate), 2C_14_H_21_N_2_O^+^·C_4_H_2_O_4_^2−^, the bufotenidine derivative 5-meth­oxy-2,*N*,*N*,*N*-tetra­methyl­tryptammonium (5-MeO-2-Me-TMT) iodide {systematic name: [2-(5-meth­oxy-2-methyl-1*H*-indol-3-yl)eth­yl]tri­methyl­aza­nium iodide}, C_15_H_23_N_2_O^+^·I^−^, and the hydrate of the same {systematic name: [2-(5-meth­oxy-2-methyl-1*H*-indol-3-yl)eth­yl]tri­methyl­aza­nium iodide monohydrate}, C_15_H_23_N_2_O^+^·I^−^·H_2_O, are reported. The structure of 5-MeO-2-Me-DMT fumarate possesses one tryptammonium cation and a half of a fumarate dianion in the asymmetric unit, linked together by N—H⋯O hydrogen bonds in infinite two-dimensional networks parallel to the (101) plane. The structure of 5-MeO-2-Me-TMT iodide possesses one tryptammonium cation and one iodide anion in the asymmetric unit. The ions are linked *via* N—H⋯I hydrogen bonds, and indoles are coupled in dimers through π–π inter­actions. The hydrate of 5-MeO-2-Me-TMT iodide possesses one tryptammonium cation, one iodide anion and one water mol­ecule in the asymmetric unit. It shows N—H⋯I and O—H⋯I hydrogen bonds that couple the tryptammonium cations into dimers.

## Abstract 2

The structures of the di­methyl­ated versions of two natural products found in toad secretions, bufotenine and bufotenidine, are reported, as well as the hydrate of 2,5-di­methyl­bufotenidine.

## Chemical context   

Bufotenine, the *N*,*N*-dimethyl analogue of serotonin, and bufotenidine, the *N*,*N*,*N*-trimethyl analogue of serotonin, were both identified in toad secretions in 1934 (Wieland *et al.*, 1934 ▸). These and other indo­alkyl­amines found in the paratoid glands of *Bufo alvarius* toads can lead to psychotropic activity in humans and other animals. Bufotenine is believed to have psychedelic properties due to its activity as a serotonin 2A agonist (Egan *et al.*, 2000 ▸). Bufotenidine (5-HTQ) is a site-selective serotonin 5-HT_3_ binder (Glennon *et al.*, 1991 ▸), and has demonstrated paralytic activity in rats (Bhattacharya & Sanyal, 1972 ▸). The best known psychedelic compound in these secretions is the *O*-methyl­ated version of bufotenine [5-meth­oxy-*N*,*N*-di­methyl­tryptamine (5-MeO-DMT)] (Spencer Jr *et al.*, 1987 ▸). Known as the ‘God Mol­ecule’, 5-MeO-DMT has been used by humans in religious ceremonies where it is traditionally administered by smoking, or vaporizing the secretions of *Bufo alvariu*s toads. 5-MeO-DMT has also been administered intra­venously, though it is inactive through oral consumption (Weil & Davis, 1994 ▸).

5-Meth­oxy-2,*N*,*N*-tri­methyl­tryptamine (5-MeO-2-Me-DMT, 2,5-dimethyl­bufotenine) was first reported in 1955, and crystallized as its picrate salt in two different forms (Shaw, 1955 ▸). A detailed synthesis of the freebase of the compound was reported by Alexander Shulgin, who also described its clinical effects on humans, with psychotropic activity occurring within an hour of oral consumption accompanied by physical stimulation (Shulgin & Shulgin, 2016 ▸). By contrast, 5-MeO-DMT is not orally active, unless consumed in combination with a mono­amine oxidase inhibitor (MAOI). The methyl­ation of the 2-position provides oral activity in 5-MeO-2-Me-DMT, likely by limiting its decomposition by mono­amine­oxidases, and also appears to reduce activity at the 5-HT_2A_ receptor, making it significantly less active than inhaled 5-MeO-DMT. Bioassays of this compound have shown it to be an agonist for the serotonin 5-HT_6_ receptor (*K_i_* = 89 n*M*) (Glennon, *et al.* 2000 ▸) and the serotonin 5-HT_7_ receptor (*K_i_* = 1,120 n*M*) (Vermeulen, *et al.* 2003 ▸).
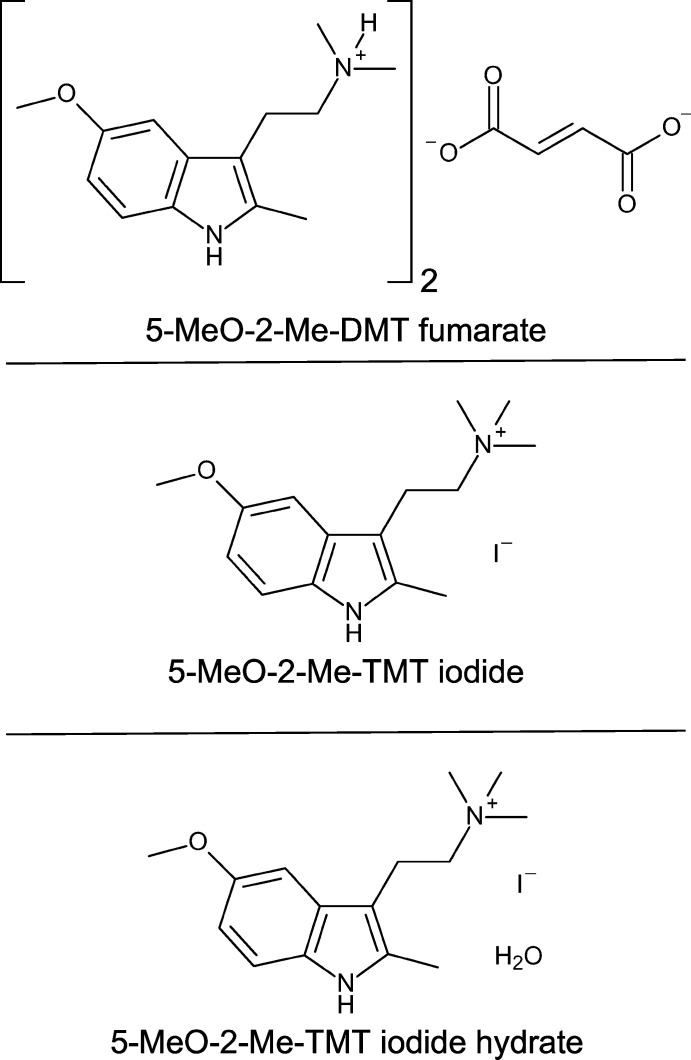


Herein we report the structure of 5-meth­oxy-2,*N*,*N*-tri­methyl­tryptammonium fumarate. We also report the synthesis of 5-meth­oxy-2,*N*,*N*,*N*-tetra­methyl­tryptammonium iodide (a bufotenidine analogue), along with its structure. Lastly, we report the structure of the first solvate of 5-meth­oxy-2,*N*,*N*,*N*-tetra­methyl­tryptammonium iodide as its hydrate.

## Structural commentary   

**Figure 1:**
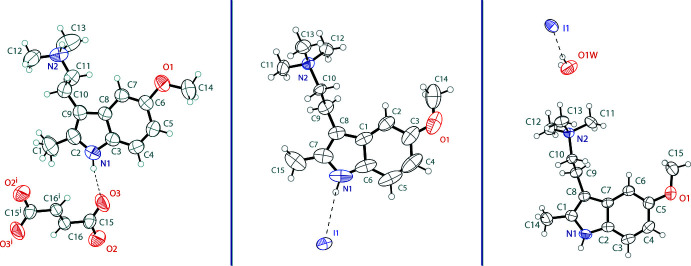
The mol­ecular structure of bis­(5-MeO-2-Me-DMT) fumarate (left), 5-MeO-2-Me-TMT iodide (center), and 5-MeO-2-Me-TMT iodide hydrate (right), showing the atomic labeling. Displacement ellipsoids are drawn at the 50% probability level. Hydrogen bonds are shown as dashed lines. Symmetry code: (i) −*x*, −*y*, 1 − *z*.

The asymmetric unit of bis­(5-meth­oxy-2,*N*,*N*-tri­methyl­tryptammonium) fumarate contains one tryptammonium cation and one half of a fumarate dianion (Fig. 1 ▸, left). The cation possesses a near planar unit containing the indole, the methyl and the meth­oxy groups, with mean deviation from planarity of 0.047 Å. The ethyl­amino group is turned away from this plane, with a C2—C9—C10—C11 torsion angle of −95.4 (2)°. The hydrogens of the 2-methyl group carbon (C1) exhibit a rotational disorder over two positions with 50% occupancy. Half of the fumarate is present in the asymmetric unit, with the other half generated by inversion. The dianion is slightly distorted from planarity with an r.m.s. deviation of 0.076 Å. The carboxyl­ate unit is delocalized with C—O distances of 1.222 (3) and 1.225 (2) Å.

The asymmetric unit of 5-meth­oxy-2,*N*,*N*,*N*-tetra­methyl­tryptammonium iodide contains one tryptammonium cation and one iodide anion (Fig. 1 ▸, center). The indole ring, methyl and meth­oxy groups of the cation are near planar, with a mean deviation from planarity of 0.050 Å. The ethyl­ammonium arm is turned away from the plane with a C7—C8—C9—C10 torsion angle of 100.9 (4)°. The asymmetric unit of its hydrate contains one tryptammonium cation, one iodide anion, and one water mol­ecule (Fig. 1 ▸, right). The tryptammonium cation is very similar to the non-hydrate, with a mean deviation from planarity of 0.043 Å for the indole ring, methyl and meth­oxy groups of the cation, and a C1—C8—C9—C10 torsion angle of 98.0 (2)°. The metrical parameters of the three structures are very similar, with the major difference observed being the elongated N—C(meth­yl) bonds in the quaternary salts.

## Supra­molecular features   

**Figure 2:**
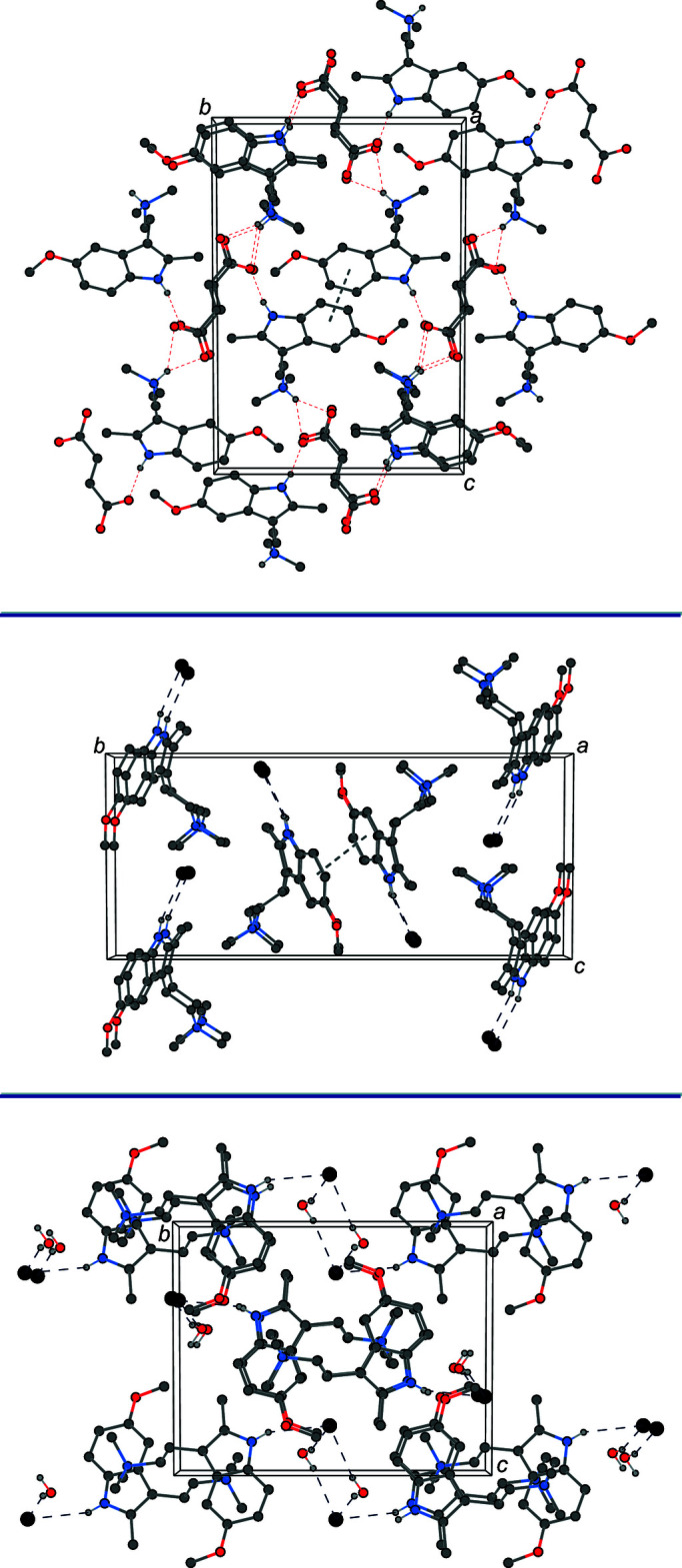
The crystal packing of bis­(5-MeO-2-Me-DMT) fumarate (top), 5-MeO-2-Me-TMT iodide (center), and 5-MeO-2-Me-TMT iodide hydrate (bottom), all shown along the *a* axis (*OLEX2*; Dolomanov *et al.*, 2009 ▸). Hydrogen bonds and π–π inter­actions are shown as dashed lines. H atoms not involved in hydrogen bonding are omitted for clarity.

In the structure of 5-MeO-2-Me-DMT fumarate, the ammonium nitro­gen exhibits a bifurcated N—H⋯(O,O) hydrogen bond with the two oxygens of a carboxyl­ate unit, and the indole nitro­gen is involved in an N—H⋯O hydrogen bond with one of the carboxyl­ate oxygens (Table 1 ▸). This series of N—H⋯O hydrogen bonds connects the ions together in an infinite two-dimensional network parallel to the (101) plane. The six-membered rings of inversion-related indoles stack with parallel slipped π–π inter­actions [inter­centroid distance = 3.9105 (15) Å, inter­planar distance = 3.7688 (19) Å, and slippage = 1.043 (3) Å]. The packing of 5-MeO-2-Me-DMT fumarate is shown at the top of Fig. 2 ▸.

In the structure of 5-MeO-2-Me-TMT iodide, the tryptammonium cation and the iodide anion are held together in the asymmetric unit *via* N*-*–H⋯I hydrogen bonds, between the indole nitro­gen and the iodide (Table 2 ▸). The six-membered rings of inversion-related indoles stack with parallel slipped π–π inter­actions [inter­centroid distance = 3.716 (3) Å, inter­planar distance = 3.488 (4) Å, and slippage = 1.282 (7) Å] that pair the tryptammonium cations together as dimers in the solid state. The packing of 5-MeO-2-Me-TMT iodide is shown in the center of Fig. 2 ▸.

In the structure of the hydrate of 5-MeO-2-Me-TMT iodide, the tryptammonium cation shows an N—H⋯I hydrogen bond between the indole nitro­gen and a symmetry-generated iodide. The water mol­ecule forms O—H⋯I hydrogen bonds with the iodide anion and another symmetry-generated iodide (Table 3 ▸). The inter­actions of two water mol­ecules and two iodide anions form diamond-shaped rings with graph-set notation 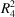
(8) (Etter *et al.*, 1990 ▸). The N—H⋯I hydrogen bonds combine with the rings to couple the tryptammonium cations together as dimers. The packing of the hydrate of 5-MeO-2-Me-TMT iodide is shown as the bottom of Fig. 2 ▸. In moving from 5-MeO-2-Me-TMT to its hydrate, the N—H⋯I inter­action is elongated as the O—H⋯I inter­actions weaken the amine–halide inter­action.

## Database survey   

The structure of bufotenine (BUFTEN: Falkenberg, 1972 ▸) and its borane adduct (OYOCIQ: Moreira *et al.*, 2015 ▸) have been reported. The unit cell of 5-MeO-DMT (QQQAGY: Bergin *et al.*, 1968 ▸) and the single crystal structure of its hydro­chloride (MOTYPT: Falkenberg & Carlström, 1971 ▸) are the other two structures reported for naturally occurring tryptamines of toads. The other simple 5-meth­oxy tryptamine whose structure is reported is the synthetic compound, 5-meth­oxy-*N*,*N*-di­allyl­tryptamine (5-MeO-DALT) (CCDC 1995802: Chadeayne *et al.*, 2020*b* ▸). The only two structures of 2-methyl­tryptamines reported are of the anti­psychotic drug oxypertine (CAGXIR: Léger *et al.*, 1983 ▸) and its bromide salt (OXYPEB10: Fillers & Hawkinson, 1978 ▸), which are used to treat schizophrenia. While the structure of bufotenidine has never been reported, the structure of four quaternary tryptammoniums have, and those are the iodide salts of 4-hy­droxy-*N*,*N*,*N*-tri­methyl­tryptamine (4-HO-TMT) and 4-acet­oxy-*N*,*N*,*N*-tri­methyl­tryptamine (4-AcO-TMT) (XUXFAA and XUXDUS: Chadeayne, Pham, Reid *et al.*, 2020 ▸), and *N*,*N*-dimethyl-*N*-*n*-propyl­tryptammonium (DMPT) and *N*,*N*-dimethyl-*N*-allyl­tryptammonium (DMALT) as their iodide salts (CCDC 2017817 and CCDC 2017818: Chadeayne *et al.*, 2020*a* ▸).

## Synthesis and crystallization   

Crystals of 5-MeO-2-Me-DMT fumarate suitable for diffraction studies were obtained from the evaporation of a methanol solution of a commercial sample (The Indole Shop). 5-MeO-2-Me-TMT iodide was synthesized when 128 mg of 5-MeO-2-Me-DMT fumarate was dissolved in 6 mL of methanol, and 6 mL of methyl­iodide was added. The mixture was refluxed under an atmosphere of nitro­gen for 12 h. The solvent was removed *in vacuo* to yield a bright-yellow powder. The powder was washed with diethyl ether to yield 127 mg of a light-yellow powder. The product was recrystallized from methanol and water to yield two different crystalline forms. The product was analyzed by ^1^H and ^13^C NMR. ^1^H NMR (400 MHz, D_2_O): δ 7.36 (*d*, *J* = 8.8 Hz, 1 H, Ar*H*), 7.04 (*d*, *J* = 2.3 Hz, 1 H, Ar*H*), 6.88 (*dd*, *J* = 8.8, 2.4 Hz, 1 H, Ar*H*), 3.88 (*s*, 3 H, OC*H*_3_), 3.44–3.40 (*m*, 2 H, C*H*_2_), 3.21 (*s*, 9 H, C*H*_3_), 3.16–3.12 (*m* 2 H, C*H*_2_), 2.38 (*s*, 3 H, C*H*_3_). ^13^C NMR (100 MHz, D_2_O): δ 152.6 (Ar*C*), 135.1 (Ar*C*), 130.3 (Ar*C*), 127.3 (Ar*C*), 111.6 (Ar*C*), 109.9 (Ar*C*), 103.0 (Ar*C*), 99.9 (Ar*C*), 65.2 (Ak*C*), 55.9 (Ak*C*), 52.4 (Ak*C*), 17.2 (Ak*C*), 10.5 (Ak*C*).

## Refinement   

Crystal data, data collection and structure refinement details are summarized in Table 4 ▸. The hydrogen atoms on the indole nitro­gen of each structure (H1) and H2 in the fumarate structure were found from a difference-Fourier map and were refined isotropically, using DFIX restraints with N—H distances of 0.87 (1) Å. Isotropic displacement parameters were set to 1.2*U*_eq_ of the parent nitro­gen atom. The hydrogen atoms on the water of the hydrate structure (H1*WA*, H1*WB*) were found from a difference-Fourier map and were refined isotropically, using a DFIX restraint with an O—H distance of 0.88 (1) Å. Isotropic displacement parameters were set to 1.5*U*_eq_ of the parent oxygen atom. All other hydrogen atoms were placed in calculated positions (C—H = 0.93-0.97 Å). Isotropic displacement parameters were set to 1.2*U*_eq_(C) or 1.5*U*_eq_(C-meth­yl). A certain number of reflections is missing from the data of all three structures. This is likely a beamstop related technical issue which could not be resolved as of yet.

## Supplementary Material

Crystal structure: contains datablock(s) umd1954c_a, umd2018f_a, umd2009b_a. DOI: 10.1107/S2056989021000803/yz2004sup1.cif

Structure factors: contains datablock(s) umd1954c_a. DOI: 10.1107/S2056989021000803/yz2004umd1954c_asup2.hkl

Structure factors: contains datablock(s) umd2018f_a. DOI: 10.1107/S2056989021000803/yz2004umd2018f_asup3.hkl

Structure factors: contains datablock(s) umd2009b_a. DOI: 10.1107/S2056989021000803/yz2004umd2009b_asup4.hkl

CCDC references: 2058145, 2058144, 2058143

Additional supporting information:  crystallographic information; 3D view; checkCIF report

## Figures and Tables

**Table 1:** Hydrogen-bond geometry (Å, °) for bis(5-MeO-2-Me-DMT) fumarate[Chem scheme1]

*D*—H⋯*A*	*D*—H	H⋯*A*	*D*⋯*A*	*D*—H⋯*A*
N1—H1⋯O3	0.87 (1)	1.95 (1)	2.810 (3)	168 (2)
N2—H2⋯O3^i^	0.88 (1)	2.18 (2)	2.892 (2)	138 (2)
N2—H2⋯O2^i^	0.88 (1)	2.00 (1)	2.837 (2)	160 (2)

Symmetry code: (i) 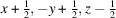.

**Table 2:** Hydrogen-bond geometry (Å, °) for 5-MeO-2-Me-TMTiodide [Chem scheme1]

*D*—H⋯*A*	*D*—H	H⋯*A*	*D*⋯*A*	*D*—H⋯*A*
N1—H1⋯I1	0.86 (1)	2.83 (2)	3.662 (3)	161 (4)

**Table 3:** Hydrogen-bond geometry (Å, °) for 5-MeO-2-Me-TMT iodide hydrate[Chem scheme1]

*D*—H⋯*A*	*D*—H	H⋯*A*	*D*⋯*A*	*D*—H⋯*A*
O1*W*—H1*WA*⋯I1^i^	0.89 (1)	2.74 (1)	3.617 (2)	168 (4)
O1*W*—H1*WB*⋯I1	0.89 (1)	2.76 (2)	3.618 (2)	164 (4)
N1—H1⋯I1^ii^	0.86 (1)	2.96 (1)	3.7416 (17)	153 (2)

Symmetry codes: (i) 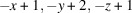; (ii) 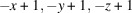.

**Table 4:** Experimental details

	bis(5-MeO-2-Me-DMT) fumarate	5-MeO-2-Me-TMT iodide	5-MeO-2-Me-TMT iodide hydrate
Crystal data			
Chemical formula	C_14_H_21_N_2_O^+^·0.5C_4_H_2_O_4_^2−^	C_15_H_23_N_2_O^+^·I^−^	C_15_H_23_N_2_O^+^·I^−^·H_2_O
*M*_r_	290.35	374.25	392.27
Crystal system, space group	Monoclinic, *P*2_1_/*n*	Monoclinic, *P*2_1_/*n*	Monoclinic, *P*2_1_/*c*
Temperature (K)	297	297	297
*a*, *b*, *c* (Å)	7.7368 (3), 12.1233 (5), 17.5528 (8)	7.5067 (8), 22.657 (3), 10.0894 (11)	10.9091 (10), 14.0910 (11), 11.4029 (10)
β (°)	102.154 (1)	97.225 (4)	100.338 (3)
*V* (Å^3^)	1609.47 (12)	1702.4 (3)	1724.4 (3)
*Z*	4	4	4
Radiation type	Mo *K*α	Mo *K*α	Mo *K*α
μ (mm^−1^)	0.08	1.88	1.86
Crystal size (mm)	0.37 × 0.24 × 0.21	0.43 × 0.20 × 0.03	0.38 × 0.22 × 0.20

Data collection			
Diffractometer	Bruker D8 Venture CMOS	Bruker D8 Venture CMOS	Bruker D8 Venture CMOS
Absorption correction	Multi-scan (*SADABS*; Bruker, 2018 ▸)	Multi-scan (*SADABS*; Bruker, 2018 ▸)	Multi-scan (*SADABS*; Bruker, 2018 ▸)
*T*_min_, *T*_max_	0.698, 0.745	0.621, 0.745	0.486, 0.562
No. of measured, independent and observed [*I* > 2σ(*I*)] reflections	36409, 3005, 2456	40959, 3207, 2875	40738, 3326, 3051
*R*_int_	0.040	0.029	0.025
(sin θ/λ)_max_ (Å^−1^)	0.611	0.611	0.618

Refinement			
*R*[*F* ^2^ > 2σ(*F* ^2^)], *wR*(*F* ^2^), *S*	0.057, 0.157, 1.03	0.028, 0.061, 1.17	0.021, 0.054, 1.10
No. of reflections	3005	3207	3326
No. of parameters	200	180	195
No. of restraints	2	1	3
H-atom treatment	H atoms treated by a mixture of independent and constrained refinement	H atoms treated by a mixture of independent and constrained refinement	H atoms treated by a mixture of independent and constrained refinement
Δρ_max_, Δρ_min_ (e Å^−3^)	0.28, −0.28	0.46, −0.80	0.40, −0.36

Computer programs: *APEX3* and *SAINT* (Bruker, 2018 ▸), *SHELXT2014* (Sheldrick, 2015*a* ▸), *SHELXL2018* (Sheldrick, 2015*b* ▸), *OLEX2* (Dolomanov *et al.*, 2009 ▸), and *publCIF* (Westrip, 2010 ▸).

## supplementary crystallographic information

### Bis{[2-(5-methoxy-2-methyl-1H-indol-3-yl)ethyl]dimethylazanium} 2E)-but-2-enedioate (umd1954c_a) Crystal data

**Table:**

C_14_H_21_N_2_O^+^·0.5C_4_H_2_O_4_^2^^−^	*F*(000) = 624
*M**_r_* = 290.35	*D*_x_ = 1.198 Mg m^−^^3^
Monoclinic, *P*2_1_/*n*	Mo *K*α radiation, λ = 0.71073 Å
*a* = 7.7368 (3) Å	Cell parameters from 9878 reflections
*b* = 12.1233 (5) Å	θ = 2.7–25.6°
*c* = 17.5528 (8) Å	µ = 0.08 mm^−^^1^
β = 102.154 (1)°	*T* = 297 K
*V* = 1609.47 (12) Å^3^	BLOCK, colourless
*Z* = 4	0.37 × 0.24 × 0.21 mm

### Bis{[2-(5-methoxy-2-methyl-1H-indol-3-yl)ethyl]dimethylazanium} 2E)-but-2-enedioate (umd1954c_a) Data collection

**Table:**

Bruker D8 Venture CMOS diffractometer	2456 reflections with *I* > 2σ(*I*)
φ and ω scans	*R*_int_ = 0.040
Absorption correction: multi-scan (SADABS; Bruker, 2018)	θ_max_ = 25.7°, θ_min_ = 2.9°
*T*_min_ = 0.698, *T*_max_ = 0.745	*h* = −9→9
36409 measured reflections	*k* = −14→14
3005 independent reflections	*l* = −21→21

### Bis{[2-(5-methoxy-2-methyl-1H-indol-3-yl)ethyl]dimethylazanium} 2E)-but-2-enedioate (umd1954c_a) Refinement

**Table:**

Refinement on *F*^2^	Hydrogen site location: mixed
Least-squares matrix: full	H atoms treated by a mixture of independent and constrained refinement
*R*[*F*^2^ > 2σ(*F*^2^)] = 0.057	*w* = 1/[σ^2^(*F*_o_^2^) + (0.0672*P*)^2^ + 0.6102*P*] where *P* = (*F*_o_^2^ + 2*F*_c_^2^)/3
*wR*(*F*^2^) = 0.157	(Δ/σ)_max_ < 0.001
*S* = 1.03	Δρ_max_ = 0.28 e Å^−^^3^
3005 reflections	Δρ_min_ = −0.28 e Å^−^^3^
200 parameters	Extinction correction: SHELXL2018 (Sheldrick, 2015b), Fc^*^=kFc[1+0.001xFc^2^λ^3^/sin(2θ)]^-1/4^
2 restraints	Extinction coefficient: 0.12 (3)
Primary atom site location: dual	

### Bis{[2-(5-methoxy-2-methyl-1H-indol-3-yl)ethyl]dimethylazanium} 2E)-but-2-enedioate (umd1954c_a) Special details

**Table:**

Geometry. All esds (except the esd in the dihedral angle between two l.s. planes) are estimated using the full covariance matrix. The cell esds are taken into account individually in the estimation of esds in distances, angles and torsion angles; correlations between esds in cell parameters are only used when they are defined by crystal symmetry. An approximate (isotropic) treatment of cell esds is used for estimating esds involving l.s. planes.

### Bis{[2-(5-methoxy-2-methyl-1H-indol-3-yl)ethyl]dimethylazanium} 2E)-but-2-enedioate (umd1954c_a) Fractional atomic coordinates and isotropic or equivalent isotropic displacement parameters (Å^2^)

**Table:**

	*x*	*y*	*z*	*U*_iso_*/*U*_eq_	Occ. (<1)
O1	0.3263 (2)	0.67305 (13)	0.37842 (10)	0.0836 (5)	
O3	0.1859 (3)	0.14716 (14)	0.58082 (10)	0.1018 (7)	
O2	0.1411 (3)	0.02997 (16)	0.66613 (8)	0.0945 (6)	
N1	0.2833 (2)	0.23477 (15)	0.44770 (11)	0.0669 (5)	
H1	0.257 (3)	0.1984 (18)	0.4864 (10)	0.080*	
N2	0.7825 (2)	0.27124 (16)	0.23889 (10)	0.0658 (5)	
H2	0.738 (3)	0.3232 (15)	0.2059 (11)	0.079*	
C1	0.3791 (4)	0.0682 (2)	0.38388 (19)	0.0938 (8)	
H1A	0.334479	0.032317	0.424551	0.141*	0.5
H1B	0.313416	0.043848	0.334105	0.141*	0.5
H1C	0.501665	0.050005	0.388708	0.141*	0.5
H1D	0.431894	0.051796	0.340358	0.141*	0.5
H1E	0.452957	0.040265	0.430804	0.141*	0.5
H1F	0.264708	0.034108	0.376201	0.141*	0.5
C2	0.3598 (3)	0.19070 (17)	0.39056 (13)	0.0656 (6)	
C3	0.2816 (2)	0.34787 (16)	0.44112 (11)	0.0560 (5)	
C4	0.2189 (3)	0.42778 (19)	0.48417 (12)	0.0659 (6)	
H4	0.167979	0.408617	0.525816	0.079*	
C5	0.2334 (3)	0.53672 (18)	0.46408 (13)	0.0667 (6)	
H5	0.193328	0.591857	0.492890	0.080*	
C6	0.3078 (3)	0.56512 (16)	0.40087 (12)	0.0605 (5)	
C7	0.3669 (2)	0.48565 (16)	0.35662 (11)	0.0557 (5)	
H7	0.413399	0.505344	0.313799	0.067*	
C8	0.3562 (2)	0.37523 (15)	0.37691 (10)	0.0515 (5)	
C9	0.4050 (2)	0.27364 (16)	0.34566 (12)	0.0574 (5)	
C10	0.4906 (3)	0.26084 (19)	0.27770 (13)	0.0665 (6)	
H10A	0.444341	0.315705	0.238481	0.080*	
H10B	0.464230	0.188392	0.254737	0.080*	
C11	0.6900 (3)	0.27476 (19)	0.30381 (12)	0.0651 (6)	
H11A	0.714481	0.344780	0.330661	0.078*	
H11B	0.735842	0.216709	0.340560	0.078*	
C12	0.7638 (4)	0.1629 (2)	0.19843 (16)	0.0978 (9)	
H12A	0.641302	0.149529	0.176090	0.147*	
H12B	0.807970	0.105647	0.235151	0.147*	
H12C	0.829966	0.163573	0.157907	0.147*	
C13	0.9701 (3)	0.2993 (3)	0.26402 (18)	0.1124 (12)	
H13A	0.981166	0.371141	0.287591	0.169*	
H13B	1.024691	0.299440	0.219710	0.169*	
H13C	1.027309	0.245802	0.301211	0.169*	
C14	0.2885 (4)	0.7581 (2)	0.42723 (17)	0.0922 (8)	
H14A	0.311697	0.828292	0.406078	0.138*	
H14B	0.361662	0.749779	0.478335	0.138*	
H14C	0.166321	0.754253	0.430504	0.138*	
C15	0.1294 (3)	0.05865 (16)	0.59833 (10)	0.0567 (5)	
C16	0.0335 (3)	−0.01511 (14)	0.53555 (10)	0.0530 (5)	
H16	0.020537	−0.088678	0.548278	0.064*	

### Bis{[2-(5-methoxy-2-methyl-1H-indol-3-yl)ethyl]dimethylazanium} 2E)-but-2-enedioate (umd1954c_a) Atomic displacement parameters (Å^2^)

**Table:**

	*U*^11^	*U*^22^	*U*^33^	*U*^12^	*U*^13^	*U*^23^
O1	0.1053 (13)	0.0551 (9)	0.0918 (11)	0.0036 (8)	0.0242 (9)	0.0046 (8)
O3	0.1565 (19)	0.0704 (10)	0.0670 (10)	−0.0486 (11)	−0.0027 (10)	−0.0076 (8)
O2	0.1179 (14)	0.1187 (15)	0.0407 (8)	−0.0287 (11)	0.0028 (8)	−0.0025 (8)
N1	0.0666 (11)	0.0622 (11)	0.0717 (11)	−0.0061 (8)	0.0140 (9)	0.0090 (8)
N2	0.0500 (9)	0.0853 (13)	0.0599 (10)	0.0160 (8)	0.0066 (7)	0.0249 (9)
C1	0.0986 (19)	0.0601 (14)	0.121 (2)	0.0031 (13)	0.0187 (16)	0.0006 (14)
C2	0.0545 (11)	0.0587 (11)	0.0790 (14)	−0.0016 (9)	0.0032 (10)	−0.0014 (10)
C3	0.0470 (9)	0.0604 (11)	0.0580 (11)	−0.0049 (8)	0.0051 (8)	0.0038 (8)
C4	0.0643 (12)	0.0782 (14)	0.0570 (11)	−0.0023 (10)	0.0166 (9)	0.0029 (10)
C5	0.0673 (13)	0.0677 (13)	0.0656 (12)	0.0062 (10)	0.0149 (10)	−0.0073 (10)
C6	0.0561 (11)	0.0571 (11)	0.0647 (12)	−0.0013 (8)	0.0042 (9)	0.0034 (9)
C7	0.0474 (9)	0.0609 (11)	0.0575 (11)	−0.0052 (8)	0.0085 (8)	0.0019 (8)
C8	0.0378 (8)	0.0581 (10)	0.0555 (10)	−0.0050 (7)	0.0027 (7)	−0.0004 (8)
C9	0.0421 (9)	0.0606 (11)	0.0671 (11)	−0.0022 (8)	0.0060 (8)	−0.0052 (9)
C10	0.0516 (11)	0.0749 (13)	0.0711 (13)	−0.0013 (9)	0.0088 (9)	−0.0118 (10)
C11	0.0517 (11)	0.0813 (14)	0.0602 (11)	0.0098 (9)	0.0067 (9)	−0.0021 (10)
C12	0.130 (2)	0.0959 (19)	0.0772 (16)	0.0241 (17)	0.0451 (16)	0.0052 (14)
C13	0.0471 (12)	0.196 (4)	0.0931 (19)	0.0094 (16)	0.0133 (12)	0.017 (2)
C14	0.106 (2)	0.0614 (13)	0.1019 (19)	0.0060 (13)	0.0050 (16)	−0.0115 (13)
C15	0.0618 (11)	0.0609 (11)	0.0447 (10)	−0.0017 (9)	0.0053 (8)	−0.0072 (8)
C16	0.0666 (11)	0.0443 (9)	0.0460 (9)	−0.0062 (8)	0.0074 (8)	0.0000 (7)

### Bis{[2-(5-methoxy-2-methyl-1H-indol-3-yl)ethyl]dimethylazanium} 2E)-but-2-enedioate (umd1954c_a) Geometric parameters (Å, º)

**Table:**

O1—C6	1.382 (2)	C5—C6	1.396 (3)
O1—C14	1.410 (3)	C6—C7	1.375 (3)
O3—C15	1.222 (2)	C7—H7	0.9300
O2—C15	1.225 (2)	C7—C8	1.392 (3)
N1—H1	0.870 (10)	C8—C9	1.431 (3)
N1—C2	1.375 (3)	C9—C10	1.490 (3)
N1—C3	1.376 (3)	C10—H10A	0.9700
N2—H2	0.876 (10)	C10—H10B	0.9700
N2—C11	1.469 (3)	C10—C11	1.523 (3)
N2—C12	1.485 (3)	C11—H11A	0.9700
N2—C13	1.465 (3)	C11—H11B	0.9700
C1—H1A	0.9600	C12—H12A	0.9600
C1—H1B	0.9600	C12—H12B	0.9600
C1—H1C	0.9600	C12—H12C	0.9600
C1—H1D	0.9600	C13—H13A	0.9600
C1—H1E	0.9600	C13—H13B	0.9600
C1—H1F	0.9600	C13—H13C	0.9600
C1—C2	1.500 (3)	C14—H14A	0.9600
C2—C9	1.368 (3)	C14—H14B	0.9600
C3—C4	1.378 (3)	C14—H14C	0.9600
C3—C8	1.410 (3)	C15—C16	1.490 (2)
C4—H4	0.9300	C16—C16^i^	1.299 (3)
C4—C5	1.378 (3)	C16—H16	0.9300
C5—H5	0.9300		
			
C6—O1—C14	118.16 (19)	C7—C8—C3	119.16 (18)
C2—N1—H1	125.5 (17)	C7—C8—C9	134.02 (18)
C2—N1—C3	108.90 (17)	C2—C9—C8	106.98 (18)
C3—N1—H1	124.8 (17)	C2—C9—C10	126.55 (19)
C11—N2—H2	107.6 (16)	C8—C9—C10	126.46 (18)
C11—N2—C12	112.47 (19)	C9—C10—H10A	109.7
C12—N2—H2	109.6 (16)	C9—C10—H10B	109.7
C13—N2—H2	105.0 (16)	C9—C10—C11	109.91 (17)
C13—N2—C11	111.93 (19)	H10A—C10—H10B	108.2
C13—N2—C12	109.9 (2)	C11—C10—H10A	109.7
H1A—C1—H1B	109.5	C11—C10—H10B	109.7
H1A—C1—H1C	109.5	N2—C11—C10	113.05 (17)
H1B—C1—H1C	109.5	N2—C11—H11A	109.0
H1D—C1—H1E	109.5	N2—C11—H11B	109.0
H1D—C1—H1F	109.5	C10—C11—H11A	109.0
H1E—C1—H1F	109.5	C10—C11—H11B	109.0
C2—C1—H1A	109.5	H11A—C11—H11B	107.8
C2—C1—H1B	109.5	N2—C12—H12A	109.5
C2—C1—H1C	109.5	N2—C12—H12B	109.5
C2—C1—H1D	109.5	N2—C12—H12C	109.5
C2—C1—H1E	109.5	H12A—C12—H12B	109.5
C2—C1—H1F	109.5	H12A—C12—H12C	109.5
N1—C2—C1	120.5 (2)	H12B—C12—H12C	109.5
C9—C2—N1	109.64 (18)	N2—C13—H13A	109.5
C9—C2—C1	129.9 (2)	N2—C13—H13B	109.5
N1—C3—C4	130.75 (19)	N2—C13—H13C	109.5
N1—C3—C8	107.66 (18)	H13A—C13—H13B	109.5
C4—C3—C8	121.57 (18)	H13A—C13—H13C	109.5
C3—C4—H4	120.8	H13B—C13—H13C	109.5
C5—C4—C3	118.46 (19)	O1—C14—H14A	109.5
C5—C4—H4	120.8	O1—C14—H14B	109.5
C4—C5—H5	119.7	O1—C14—H14C	109.5
C4—C5—C6	120.6 (2)	H14A—C14—H14B	109.5
C6—C5—H5	119.7	H14A—C14—H14C	109.5
O1—C6—C5	122.99 (19)	H14B—C14—H14C	109.5
C7—C6—O1	115.82 (18)	O3—C15—O2	122.43 (19)
C7—C6—C5	121.19 (19)	O3—C15—C16	119.32 (17)
C6—C7—H7	120.5	O2—C15—C16	118.16 (18)
C6—C7—C8	118.98 (18)	C15—C16—H16	117.4
C8—C7—H7	120.5	C16^i^—C16—C15	125.2 (2)
C3—C8—C9	106.82 (17)	C16^i^—C16—H16	117.4
			
O1—C6—C7—C8	178.56 (17)	C3—C8—C9—C10	−179.36 (17)
O3—C15—C16—C16^i^	−17.2 (4)	C4—C3—C8—C7	0.1 (3)
O2—C15—C16—C16^i^	159.4 (3)	C4—C3—C8—C9	−179.39 (17)
N1—C2—C9—C8	0.7 (2)	C4—C5—C6—O1	−179.70 (19)
N1—C2—C9—C10	−179.92 (18)	C4—C5—C6—C7	0.6 (3)
N1—C3—C4—C5	−179.6 (2)	C5—C6—C7—C8	−1.7 (3)
N1—C3—C8—C7	178.78 (16)	C6—C7—C8—C3	1.4 (3)
N1—C3—C8—C9	−0.70 (19)	C6—C7—C8—C9	−179.32 (18)
C1—C2—C9—C8	179.4 (2)	C7—C8—C9—C2	−179.38 (19)
C1—C2—C9—C10	−1.3 (3)	C7—C8—C9—C10	1.3 (3)
C2—N1—C3—C4	179.7 (2)	C8—C3—C4—C5	−1.2 (3)
C2—N1—C3—C8	1.2 (2)	C8—C9—C10—C11	83.8 (2)
C2—C9—C10—C11	−95.4 (2)	C9—C10—C11—N2	−175.81 (18)
C3—N1—C2—C1	−180.0 (2)	C12—N2—C11—C10	−63.3 (2)
C3—N1—C2—C9	−1.2 (2)	C13—N2—C11—C10	172.4 (2)
C3—C4—C5—C6	0.9 (3)	C14—O1—C6—C5	8.0 (3)
C3—C8—C9—C2	0.0 (2)	C14—O1—C6—C7	−172.3 (2)

Symmetry code: (i) −*x*, −*y*, −*z*+1.

### Bis{[2-(5-methoxy-2-methyl-1H-indol-3-yl)ethyl]dimethylazanium} 2E)-but-2-enedioate (umd1954c_a) Hydrogen-bond geometry (Å, º)

**Table:**

*D*—H···*A*	*D*—H	H···*A*	*D*···*A*	*D*—H···*A*
N1—H1···O3	0.87 (1)	1.95 (1)	2.810 (3)	168 (2)
N2—H2···O3^ii^	0.88 (1)	2.18 (2)	2.892 (2)	138 (2)
N2—H2···O2^ii^	0.88 (1)	2.00 (1)	2.837 (2)	160 (2)

Symmetry code: (ii) *x*+1/2, −*y*+1/2, *z*−1/2.

### [2-(5-Methoxy-2-methyl-1H-indol-3-yl)ethyl]trimethylazanium iodide (umd2018f_a) Crystal data

**Table:**

C_15_H_23_N_2_O^+^·I^−^	*F*(000) = 752
*M**_r_* = 374.25	*D*_x_ = 1.460 Mg m^−^^3^
Monoclinic, *P*2_1_/*n*	Mo *K*α radiation, λ = 0.71073 Å
*a* = 7.5067 (8) Å	Cell parameters from 9773 reflections
*b* = 22.657 (3) Å	θ = 2.9–25.7°
*c* = 10.0894 (11) Å	µ = 1.88 mm^−^^1^
β = 97.225 (4)°	*T* = 297 K
*V* = 1702.4 (3) Å^3^	PLATE, colourless
*Z* = 4	0.43 × 0.20 × 0.03 mm

### [2-(5-Methoxy-2-methyl-1H-indol-3-yl)ethyl]trimethylazanium iodide (umd2018f_a) Data collection

**Table:**

Bruker D8 Venture CMOS diffractometer	2875 reflections with *I* > 2σ(*I*)
φ and ω scans	*R*_int_ = 0.029
Absorption correction: multi-scan (SADABS; Bruker, 2018)	θ_max_ = 25.7°, θ_min_ = 2.7°
*T*_min_ = 0.621, *T*_max_ = 0.745	*h* = −9→8
40959 measured reflections	*k* = −27→27
3207 independent reflections	*l* = −12→12

### [2-(5-Methoxy-2-methyl-1H-indol-3-yl)ethyl]trimethylazanium iodide (umd2018f_a) Refinement

**Table:**

Refinement on *F*^2^	Primary atom site location: dual
Least-squares matrix: full	Hydrogen site location: mixed
*R*[*F*^2^ > 2σ(*F*^2^)] = 0.028	H atoms treated by a mixture of independent and constrained refinement
*wR*(*F*^2^) = 0.061	*w* = 1/[σ^2^(*F*_o_^2^) + 2.306*P*] where *P* = (*F*_o_^2^ + 2*F*_c_^2^)/3
*S* = 1.17	(Δ/σ)_max_ = 0.001
3207 reflections	Δρ_max_ = 0.46 e Å^−^^3^
180 parameters	Δρ_min_ = −0.80 e Å^−^^3^
1 restraint	

### [2-(5-Methoxy-2-methyl-1H-indol-3-yl)ethyl]trimethylazanium iodide (umd2018f_a) Special details

**Table:**

Geometry. All esds (except the esd in the dihedral angle between two l.s. planes) are estimated using the full covariance matrix. The cell esds are taken into account individually in the estimation of esds in distances, angles and torsion angles; correlations between esds in cell parameters are only used when they are defined by crystal symmetry. An approximate (isotropic) treatment of cell esds is used for estimating esds involving l.s. planes.

### [2-(5-Methoxy-2-methyl-1H-indol-3-yl)ethyl]trimethylazanium iodide (umd2018f_a) Fractional atomic coordinates and isotropic or equivalent isotropic displacement parameters (Å^2^)

**Table:**

	*x*	*y*	*z*	*U*_iso_*/*U*_eq_	
I1	0.43011 (3)	0.33522 (2)	0.91956 (2)	0.05592 (9)	
O1	0.1841 (4)	0.49375 (15)	0.1758 (4)	0.1013 (11)	
N1	0.5763 (6)	0.39020 (16)	0.6094 (3)	0.0793 (11)	
H1	0.563 (6)	0.3830 (19)	0.6915 (17)	0.095*	
N2	0.9563 (3)	0.31308 (11)	0.1516 (3)	0.0481 (6)	
C1	0.5377 (4)	0.41400 (14)	0.3926 (4)	0.0538 (8)	
C2	0.4496 (5)	0.43986 (15)	0.2768 (4)	0.0596 (9)	
H2	0.499719	0.438575	0.197133	0.072*	
C3	0.2864 (5)	0.46743 (17)	0.2829 (5)	0.0763 (12)	
C4	0.2127 (6)	0.4695 (2)	0.4038 (6)	0.0954 (18)	
H4	0.103476	0.488605	0.406264	0.114*	
C5	0.2955 (6)	0.4446 (2)	0.5172 (6)	0.0921 (17)	
H5	0.244398	0.446392	0.596395	0.110*	
C6	0.4595 (6)	0.41628 (17)	0.5120 (4)	0.0687 (11)	
C7	0.7278 (6)	0.37181 (16)	0.5563 (4)	0.0676 (10)	
C8	0.7079 (5)	0.38575 (14)	0.4236 (3)	0.0529 (8)	
C9	0.8472 (4)	0.37817 (15)	0.3310 (3)	0.0551 (8)	
H9A	0.964663	0.375543	0.383112	0.066*	
H9B	0.846742	0.412634	0.273907	0.066*	
C10	0.8157 (4)	0.32358 (13)	0.2446 (3)	0.0422 (6)	
H10A	0.699126	0.326878	0.191522	0.051*	
H10B	0.812240	0.289466	0.302324	0.051*	
C11	1.1387 (4)	0.30522 (18)	0.2286 (4)	0.0657 (10)	
H11A	1.172879	0.340747	0.277101	0.099*	
H11B	1.224149	0.296830	0.167952	0.099*	
H11C	1.135847	0.273038	0.290226	0.099*	
C12	0.9611 (5)	0.36360 (16)	0.0563 (3)	0.0615 (9)	
H12A	1.000613	0.398594	0.104965	0.092*	
H12B	0.843013	0.369957	0.009750	0.092*	
H12C	1.042595	0.354613	−0.006982	0.092*	
C13	0.9054 (6)	0.25820 (16)	0.0735 (4)	0.0669 (10)	
H13A	0.991867	0.250697	0.013065	0.100*	
H13B	0.788748	0.263219	0.023630	0.100*	
H13C	0.902829	0.225467	0.133626	0.100*	
C14	0.2614 (7)	0.4976 (2)	0.0551 (6)	0.1045 (17)	
H14A	0.175989	0.514516	−0.013254	0.157*	
H14B	0.293959	0.458831	0.028158	0.157*	
H14C	0.366534	0.522046	0.068418	0.157*	
C15	0.8829 (8)	0.3443 (2)	0.6421 (5)	0.1011 (16)	
H15A	0.928146	0.311742	0.595332	0.152*	
H15B	0.843973	0.330378	0.723583	0.152*	
H15C	0.976182	0.373088	0.662330	0.152*	

### [2-(5-Methoxy-2-methyl-1H-indol-3-yl)ethyl]trimethylazanium iodide (umd2018f_a) Atomic displacement parameters (Å^2^)

**Table:**

	*U*^11^	*U*^22^	*U*^33^	*U*^12^	*U*^13^	*U*^23^
I1	0.05627 (14)	0.06367 (15)	0.05083 (13)	−0.00004 (11)	0.01850 (9)	−0.00788 (10)
O1	0.0650 (18)	0.091 (2)	0.143 (3)	0.0200 (17)	−0.007 (2)	−0.037 (2)
N1	0.113 (3)	0.077 (2)	0.056 (2)	−0.041 (2)	0.040 (2)	−0.0190 (18)
N2	0.0462 (14)	0.0540 (15)	0.0465 (14)	0.0096 (12)	0.0151 (11)	0.0100 (12)
C1	0.0515 (19)	0.0489 (18)	0.065 (2)	−0.0174 (15)	0.0235 (16)	−0.0221 (16)
C2	0.0524 (19)	0.055 (2)	0.074 (2)	−0.0069 (16)	0.0175 (17)	−0.0222 (18)
C3	0.051 (2)	0.062 (2)	0.116 (4)	−0.0094 (18)	0.014 (2)	−0.040 (2)
C4	0.055 (2)	0.083 (3)	0.155 (5)	−0.022 (2)	0.041 (3)	−0.071 (3)
C5	0.078 (3)	0.090 (3)	0.122 (4)	−0.044 (3)	0.062 (3)	−0.066 (3)
C6	0.074 (3)	0.064 (2)	0.075 (3)	−0.035 (2)	0.039 (2)	−0.035 (2)
C7	0.089 (3)	0.056 (2)	0.059 (2)	−0.027 (2)	0.016 (2)	−0.0099 (17)
C8	0.059 (2)	0.0481 (18)	0.0541 (19)	−0.0156 (15)	0.0167 (15)	−0.0122 (15)
C9	0.0495 (18)	0.0537 (19)	0.064 (2)	−0.0099 (15)	0.0136 (15)	−0.0049 (16)
C10	0.0345 (14)	0.0490 (17)	0.0465 (16)	0.0011 (12)	0.0180 (12)	0.0028 (13)
C11	0.0437 (18)	0.082 (3)	0.073 (2)	0.0165 (17)	0.0138 (16)	0.023 (2)
C12	0.060 (2)	0.072 (2)	0.057 (2)	0.0108 (17)	0.0217 (16)	0.0255 (17)
C13	0.086 (3)	0.060 (2)	0.057 (2)	0.0162 (19)	0.0179 (19)	−0.0047 (17)
C14	0.091 (4)	0.066 (3)	0.150 (5)	0.013 (3)	−0.011 (4)	0.014 (3)
C15	0.132 (4)	0.096 (4)	0.071 (3)	−0.022 (3)	−0.006 (3)	0.013 (3)

### [2-(5-Methoxy-2-methyl-1H-indol-3-yl)ethyl]trimethylazanium iodide (umd2018f_a) Geometric parameters (Å, º)

**Table:**

O1—C3	1.380 (5)	C8—C9	1.497 (4)
O1—C14	1.416 (6)	C9—H9A	0.9700
N1—H1	0.863 (10)	C9—H9B	0.9700
N1—C6	1.367 (6)	C9—C10	1.514 (4)
N1—C7	1.381 (5)	C10—H10A	0.9700
N2—C10	1.517 (3)	C10—H10B	0.9700
N2—C11	1.497 (4)	C11—H11A	0.9600
N2—C12	1.498 (4)	C11—H11B	0.9600
N2—C13	1.496 (4)	C11—H11C	0.9600
C1—C2	1.397 (5)	C12—H12A	0.9600
C1—C6	1.406 (5)	C12—H12B	0.9600
C1—C8	1.428 (5)	C12—H12C	0.9600
C2—H2	0.9300	C13—H13A	0.9600
C2—C3	1.383 (5)	C13—H13B	0.9600
C3—C4	1.403 (7)	C13—H13C	0.9600
C4—H4	0.9300	C14—H14A	0.9600
C4—C5	1.354 (7)	C14—H14B	0.9600
C5—H5	0.9300	C14—H14C	0.9600
C5—C6	1.396 (6)	C15—H15A	0.9600
C7—C8	1.365 (5)	C15—H15B	0.9600
C7—C15	1.497 (6)	C15—H15C	0.9600
			
C3—O1—C14	116.9 (3)	C10—C9—H9A	109.1
C6—N1—H1	129 (3)	C10—C9—H9B	109.1
C6—N1—C7	109.6 (3)	N2—C10—H10A	108.6
C7—N1—H1	121 (3)	N2—C10—H10B	108.6
C11—N2—C10	111.0 (2)	C9—C10—N2	114.5 (2)
C11—N2—C12	109.3 (3)	C9—C10—H10A	108.6
C12—N2—C10	110.6 (2)	C9—C10—H10B	108.6
C13—N2—C10	107.8 (2)	H10A—C10—H10B	107.6
C13—N2—C11	109.3 (3)	N2—C11—H11A	109.5
C13—N2—C12	108.8 (3)	N2—C11—H11B	109.5
C2—C1—C6	119.8 (4)	N2—C11—H11C	109.5
C2—C1—C8	133.4 (3)	H11A—C11—H11B	109.5
C6—C1—C8	106.7 (4)	H11A—C11—H11C	109.5
C1—C2—H2	120.6	H11B—C11—H11C	109.5
C3—C2—C1	118.8 (4)	N2—C12—H12A	109.5
C3—C2—H2	120.6	N2—C12—H12B	109.5
O1—C3—C2	124.7 (4)	N2—C12—H12C	109.5
O1—C3—C4	115.2 (4)	H12A—C12—H12B	109.5
C2—C3—C4	120.1 (5)	H12A—C12—H12C	109.5
C3—C4—H4	119.0	H12B—C12—H12C	109.5
C5—C4—C3	122.1 (4)	N2—C13—H13A	109.5
C5—C4—H4	119.0	N2—C13—H13B	109.5
C4—C5—H5	120.9	N2—C13—H13C	109.5
C4—C5—C6	118.3 (4)	H13A—C13—H13B	109.5
C6—C5—H5	120.9	H13A—C13—H13C	109.5
N1—C6—C1	107.6 (4)	H13B—C13—H13C	109.5
N1—C6—C5	131.4 (4)	O1—C14—H14A	109.5
C5—C6—C1	120.9 (5)	O1—C14—H14B	109.5
N1—C7—C15	121.3 (4)	O1—C14—H14C	109.5
C8—C7—N1	108.5 (4)	H14A—C14—H14B	109.5
C8—C7—C15	130.1 (4)	H14A—C14—H14C	109.5
C1—C8—C9	126.2 (3)	H14B—C14—H14C	109.5
C7—C8—C1	107.6 (3)	C7—C15—H15A	109.5
C7—C8—C9	125.9 (4)	C7—C15—H15B	109.5
C8—C9—H9A	109.1	C7—C15—H15C	109.5
C8—C9—H9B	109.1	H15A—C15—H15B	109.5
C8—C9—C10	112.5 (2)	H15A—C15—H15C	109.5
H9A—C9—H9B	107.8	H15B—C15—H15C	109.5
			
O1—C3—C4—C5	178.7 (4)	C6—C1—C8—C7	0.3 (4)
N1—C7—C8—C1	0.1 (4)	C6—C1—C8—C9	−174.2 (3)
N1—C7—C8—C9	174.5 (3)	C7—N1—C6—C1	0.6 (4)
C1—C2—C3—O1	−178.8 (3)	C7—N1—C6—C5	−175.7 (4)
C1—C2—C3—C4	0.5 (5)	C7—C8—C9—C10	100.9 (4)
C1—C8—C9—C10	−85.7 (4)	C8—C1—C2—C3	−175.7 (3)
C2—C1—C6—N1	−177.4 (3)	C8—C1—C6—N1	−0.5 (4)
C2—C1—C6—C5	−0.7 (5)	C8—C1—C6—C5	176.2 (3)
C2—C1—C8—C7	176.6 (3)	C8—C9—C10—N2	−178.5 (3)
C2—C1—C8—C9	2.1 (6)	C11—N2—C10—C9	60.6 (3)
C2—C3—C4—C5	−0.7 (6)	C12—N2—C10—C9	−60.8 (4)
C3—C4—C5—C6	0.2 (6)	C13—N2—C10—C9	−179.7 (3)
C4—C5—C6—N1	176.4 (4)	C14—O1—C3—C2	−6.5 (6)
C4—C5—C6—C1	0.5 (6)	C14—O1—C3—C4	174.1 (4)
C6—N1—C7—C8	−0.4 (4)	C15—C7—C8—C1	−177.0 (4)
C6—N1—C7—C15	177.0 (3)	C15—C7—C8—C9	−2.6 (6)
C6—C1—C2—C3	0.2 (5)		

### [2-(5-Methoxy-2-methyl-1H-indol-3-yl)ethyl]trimethylazanium iodide (umd2018f_a) Hydrogen-bond geometry (Å, º)

**Table:**

*D*—H···*A*	*D*—H	H···*A*	*D*···*A*	*D*—H···*A*
N1—H1···I1	0.86 (1)	2.83 (2)	3.662 (3)	161 (4)

### [2-(5-Methoxy-2-methyl-1H-indol-3-yl)ethyl]trimethylazanium iodide monohydrate (umd2009b_a) Crystal data

**Table:**

C_15_H_23_N_2_O^+^·I^−^·H_2_O	*F*(000) = 792
*M**_r_* = 392.27	*D*_x_ = 1.511 Mg m^−^^3^
Monoclinic, *P*2_1_/*c*	Mo *K*α radiation, λ = 0.71073 Å
*a* = 10.9091 (10) Å	Cell parameters from 9808 reflections
*b* = 14.0910 (11) Å	θ = 2.8–26.0°
*c* = 11.4029 (10) Å	µ = 1.86 mm^−^^1^
β = 100.338 (3)°	*T* = 297 K
*V* = 1724.4 (3) Å^3^	BLOCK, colourless
*Z* = 4	0.38 × 0.22 × 0.20 mm

### [2-(5-Methoxy-2-methyl-1H-indol-3-yl)ethyl]trimethylazanium iodide monohydrate (umd2009b_a) Data collection

**Table:**

Bruker D8 Venture CMOS diffractometer	3051 reflections with *I* > 2σ(*I*)
φ and ω scans	*R*_int_ = 0.025
Absorption correction: multi-scan (SADABS; Bruker, 2018)	θ_max_ = 26.1°, θ_min_ = 3.8°
*T*_min_ = 0.486, *T*_max_ = 0.562	*h* = −13→13
40738 measured reflections	*k* = −17→17
3326 independent reflections	*l* = −14→14

### [2-(5-Methoxy-2-methyl-1H-indol-3-yl)ethyl]trimethylazanium iodide monohydrate (umd2009b_a) Refinement

**Table:**

Refinement on *F*^2^	Primary atom site location: dual
Least-squares matrix: full	Hydrogen site location: mixed
*R*[*F*^2^ > 2σ(*F*^2^)] = 0.021	H atoms treated by a mixture of independent and constrained refinement
*wR*(*F*^2^) = 0.054	*w* = 1/[σ^2^(*F*_o_^2^) + (0.0198*P*)^2^ + 0.9135*P*] where *P* = (*F*_o_^2^ + 2*F*_c_^2^)/3
*S* = 1.10	(Δ/σ)_max_ = 0.002
3326 reflections	Δρ_max_ = 0.40 e Å^−^^3^
195 parameters	Δρ_min_ = −0.36 e Å^−^^3^
3 restraints	

### [2-(5-Methoxy-2-methyl-1H-indol-3-yl)ethyl]trimethylazanium iodide monohydrate (umd2009b_a) Special details

**Table:**

Geometry. All esds (except the esd in the dihedral angle between two l.s. planes) are estimated using the full covariance matrix. The cell esds are taken into account individually in the estimation of esds in distances, angles and torsion angles; correlations between esds in cell parameters are only used when they are defined by crystal symmetry. An approximate (isotropic) treatment of cell esds is used for estimating esds involving l.s. planes.

### [2-(5-Methoxy-2-methyl-1H-indol-3-yl)ethyl]trimethylazanium iodide monohydrate (umd2009b_a) Fractional atomic coordinates and isotropic or equivalent isotropic displacement parameters (Å^2^)

**Table:**

	*x*	*y*	*z*	*U*_iso_*/*U*_eq_	
I1	0.66404 (2)	0.98962 (2)	0.30669 (2)	0.05629 (7)	
O1	−0.06415 (16)	0.35370 (12)	0.19831 (14)	0.0645 (4)	
O1W	0.3969 (2)	0.90251 (15)	0.4217 (2)	0.0846 (6)	
H1WA	0.384 (4)	0.937 (3)	0.483 (2)	0.127*	
H1WB	0.465 (2)	0.931 (3)	0.407 (4)	0.127*	
N1	0.21469 (17)	0.25708 (12)	0.63123 (17)	0.0499 (4)	
H1	0.231 (2)	0.2044 (11)	0.6685 (19)	0.060*	
N2	0.35006 (14)	0.65530 (10)	0.53057 (13)	0.0379 (3)	
C1	0.25645 (18)	0.34390 (14)	0.67593 (18)	0.0445 (4)	
C2	0.14222 (18)	0.26828 (13)	0.52036 (18)	0.0432 (4)	
C3	0.0771 (2)	0.20291 (14)	0.4425 (2)	0.0544 (5)	
H3	0.078699	0.138686	0.461520	0.065*	
C4	0.0105 (2)	0.23508 (15)	0.3370 (2)	0.0551 (5)	
H4	−0.034166	0.192097	0.283837	0.066*	
C5	0.00816 (18)	0.33197 (15)	0.30727 (18)	0.0468 (4)	
C6	0.07209 (17)	0.39779 (13)	0.38389 (17)	0.0414 (4)	
H6	0.070468	0.461808	0.363818	0.050*	
C7	0.14001 (16)	0.36583 (12)	0.49342 (17)	0.0378 (4)	
C8	0.21289 (16)	0.41246 (13)	0.59414 (17)	0.0391 (4)	
C9	0.23415 (19)	0.51747 (13)	0.60723 (19)	0.0421 (4)	
H9A	0.253828	0.533648	0.691189	0.051*	
H9B	0.158531	0.550853	0.572574	0.051*	
C10	0.33991 (16)	0.54890 (12)	0.54616 (16)	0.0354 (4)	
H10A	0.417725	0.525925	0.592161	0.042*	
H10B	0.329195	0.519225	0.468226	0.042*	
C11	0.2417 (2)	0.69182 (16)	0.4424 (2)	0.0589 (6)	
H11A	0.254727	0.757586	0.426376	0.088*	
H11B	0.166686	0.685097	0.474590	0.088*	
H11C	0.234330	0.656169	0.369714	0.088*	
C12	0.3573 (2)	0.70577 (15)	0.6465 (2)	0.0548 (5)	
H12A	0.370823	0.772233	0.635447	0.082*	
H12B	0.425002	0.680458	0.703390	0.082*	
H12C	0.280615	0.697125	0.675441	0.082*	
C13	0.4675 (2)	0.67358 (16)	0.4820 (2)	0.0553 (5)	
H13A	0.475864	0.740446	0.469415	0.083*	
H13B	0.463369	0.640606	0.407765	0.083*	
H13C	0.538030	0.651344	0.538002	0.083*	
C14	0.3351 (2)	0.35188 (18)	0.7972 (2)	0.0608 (6)	
H14A	0.313836	0.408931	0.835009	0.091*	
H14B	0.421438	0.353824	0.790177	0.091*	
H14C	0.320503	0.297993	0.844379	0.091*	
C15	−0.0569 (2)	0.44733 (19)	0.1541 (2)	0.0610 (6)	
H15A	−0.107259	0.451967	0.076067	0.092*	
H15B	0.028092	0.461878	0.149538	0.092*	
H15C	−0.086653	0.491467	0.206657	0.092*	

### [2-(5-Methoxy-2-methyl-1H-indol-3-yl)ethyl]trimethylazanium iodide monohydrate (umd2009b_a) Atomic displacement parameters (Å^2^)

**Table:**

	*U*^11^	*U*^22^	*U*^33^	*U*^12^	*U*^13^	*U*^23^
I1	0.05807 (11)	0.05975 (11)	0.05273 (10)	0.01395 (6)	0.01446 (7)	0.00736 (6)
O1	0.0638 (10)	0.0650 (10)	0.0573 (9)	−0.0043 (8)	−0.0091 (8)	−0.0007 (8)
O1W	0.1088 (17)	0.0667 (12)	0.0840 (14)	−0.0251 (11)	0.0323 (12)	−0.0142 (10)
N1	0.0541 (10)	0.0356 (8)	0.0590 (11)	0.0022 (7)	0.0080 (8)	0.0119 (7)
N2	0.0402 (8)	0.0314 (7)	0.0412 (8)	0.0007 (6)	0.0052 (6)	0.0012 (6)
C1	0.0367 (9)	0.0451 (10)	0.0524 (11)	0.0013 (8)	0.0102 (8)	0.0061 (8)
C2	0.0436 (10)	0.0346 (9)	0.0530 (11)	0.0008 (8)	0.0134 (8)	0.0047 (8)
C3	0.0621 (13)	0.0328 (9)	0.0699 (14)	−0.0055 (9)	0.0155 (11)	−0.0010 (9)
C4	0.0550 (12)	0.0449 (11)	0.0643 (14)	−0.0095 (9)	0.0076 (10)	−0.0119 (10)
C5	0.0408 (10)	0.0510 (11)	0.0488 (11)	−0.0003 (8)	0.0086 (8)	−0.0020 (9)
C6	0.0381 (9)	0.0365 (9)	0.0507 (11)	0.0002 (7)	0.0110 (8)	0.0038 (8)
C7	0.0340 (9)	0.0328 (9)	0.0488 (10)	−0.0005 (7)	0.0135 (8)	0.0014 (7)
C8	0.0358 (9)	0.0367 (9)	0.0468 (10)	−0.0016 (7)	0.0128 (8)	0.0015 (7)
C9	0.0436 (10)	0.0358 (9)	0.0493 (11)	−0.0011 (7)	0.0146 (8)	−0.0033 (8)
C10	0.0370 (9)	0.0282 (8)	0.0411 (9)	0.0017 (7)	0.0074 (7)	−0.0007 (7)
C11	0.0586 (13)	0.0497 (12)	0.0625 (13)	0.0088 (10)	−0.0054 (11)	0.0158 (10)
C12	0.0716 (14)	0.0403 (10)	0.0530 (12)	−0.0058 (10)	0.0124 (10)	−0.0114 (9)
C13	0.0546 (12)	0.0456 (11)	0.0698 (14)	−0.0080 (9)	0.0220 (11)	0.0031 (10)
C14	0.0528 (13)	0.0664 (14)	0.0596 (13)	−0.0004 (11)	0.0003 (10)	0.0108 (11)
C15	0.0559 (13)	0.0749 (16)	0.0513 (12)	0.0025 (11)	0.0071 (10)	0.0113 (11)

### [2-(5-Methoxy-2-methyl-1H-indol-3-yl)ethyl]trimethylazanium iodide monohydrate (umd2009b_a) Geometric parameters (Å, º)

**Table:**

O1—C5	1.381 (3)	C7—C8	1.434 (3)
O1—C15	1.420 (3)	C8—C9	1.501 (2)
O1W—H1WA	0.888 (10)	C9—H9A	0.9700
O1W—H1WB	0.886 (10)	C9—H9B	0.9700
N1—H1	0.858 (10)	C9—C10	1.517 (3)
N1—C1	1.371 (3)	C10—H10A	0.9700
N1—C2	1.375 (3)	C10—H10B	0.9700
N2—C10	1.516 (2)	C11—H11A	0.9600
N2—C11	1.499 (2)	C11—H11B	0.9600
N2—C12	1.491 (2)	C11—H11C	0.9600
N2—C13	1.506 (2)	C12—H12A	0.9600
C1—C8	1.367 (3)	C12—H12B	0.9600
C1—C14	1.495 (3)	C12—H12C	0.9600
C2—C3	1.384 (3)	C13—H13A	0.9600
C2—C7	1.408 (2)	C13—H13B	0.9600
C3—H3	0.9300	C13—H13C	0.9600
C3—C4	1.367 (3)	C14—H14A	0.9600
C4—H4	0.9300	C14—H14B	0.9600
C4—C5	1.406 (3)	C14—H14C	0.9600
C5—C6	1.375 (3)	C15—H15A	0.9600
C6—H6	0.9300	C15—H15B	0.9600
C6—C7	1.407 (3)	C15—H15C	0.9600
			
C5—O1—C15	117.80 (17)	C10—C9—H9A	109.4
H1WA—O1W—H1WB	99 (4)	C10—C9—H9B	109.4
C1—N1—H1	124.2 (17)	N2—C10—C9	114.85 (14)
C1—N1—C2	109.71 (16)	N2—C10—H10A	108.6
C2—N1—H1	126.1 (17)	N2—C10—H10B	108.6
C11—N2—C10	110.66 (15)	C9—C10—H10A	108.6
C11—N2—C13	108.37 (17)	C9—C10—H10B	108.6
C12—N2—C10	111.16 (14)	H10A—C10—H10B	107.5
C12—N2—C11	109.93 (17)	N2—C11—H11A	109.5
C12—N2—C13	109.35 (16)	N2—C11—H11B	109.5
C13—N2—C10	107.28 (14)	N2—C11—H11C	109.5
N1—C1—C14	120.53 (18)	H11A—C11—H11B	109.5
C8—C1—N1	108.97 (17)	H11A—C11—H11C	109.5
C8—C1—C14	130.49 (19)	H11B—C11—H11C	109.5
N1—C2—C3	131.14 (18)	N2—C12—H12A	109.5
N1—C2—C7	107.29 (17)	N2—C12—H12B	109.5
C3—C2—C7	121.55 (19)	N2—C12—H12C	109.5
C2—C3—H3	120.8	H12A—C12—H12B	109.5
C4—C3—C2	118.35 (19)	H12A—C12—H12C	109.5
C4—C3—H3	120.8	H12B—C12—H12C	109.5
C3—C4—H4	119.4	N2—C13—H13A	109.5
C3—C4—C5	121.18 (19)	N2—C13—H13B	109.5
C5—C4—H4	119.4	N2—C13—H13C	109.5
O1—C5—C4	114.49 (19)	H13A—C13—H13B	109.5
C6—C5—O1	124.38 (19)	H13A—C13—H13C	109.5
C6—C5—C4	121.12 (19)	H13B—C13—H13C	109.5
C5—C6—H6	120.8	C1—C14—H14A	109.5
C5—C6—C7	118.31 (17)	C1—C14—H14B	109.5
C7—C6—H6	120.8	C1—C14—H14C	109.5
C2—C7—C8	106.70 (17)	H14A—C14—H14B	109.5
C6—C7—C2	119.47 (17)	H14A—C14—H14C	109.5
C6—C7—C8	133.83 (17)	H14B—C14—H14C	109.5
C1—C8—C7	107.31 (16)	O1—C15—H15A	109.5
C1—C8—C9	126.88 (18)	O1—C15—H15B	109.5
C7—C8—C9	125.80 (17)	O1—C15—H15C	109.5
C8—C9—H9A	109.4	H15A—C15—H15B	109.5
C8—C9—H9B	109.4	H15A—C15—H15C	109.5
C8—C9—C10	111.03 (15)	H15B—C15—H15C	109.5
H9A—C9—H9B	108.0		
			
O1—C5—C6—C7	178.59 (18)	C3—C4—C5—C6	−0.6 (3)
N1—C1—C8—C7	0.1 (2)	C4—C5—C6—C7	−0.1 (3)
N1—C1—C8—C9	178.83 (17)	C5—C6—C7—C2	1.0 (3)
N1—C2—C3—C4	179.1 (2)	C5—C6—C7—C8	−177.98 (19)
N1—C2—C7—C6	179.87 (17)	C6—C7—C8—C1	179.6 (2)
N1—C2—C7—C8	−0.9 (2)	C6—C7—C8—C9	0.8 (3)
C1—N1—C2—C3	−177.8 (2)	C7—C2—C3—C4	0.5 (3)
C1—N1—C2—C7	1.0 (2)	C7—C8—C9—C10	−83.5 (2)
C1—C8—C9—C10	98.0 (2)	C8—C9—C10—N2	167.09 (15)
C2—N1—C1—C8	−0.7 (2)	C11—N2—C10—C9	−68.4 (2)
C2—N1—C1—C14	178.50 (19)	C12—N2—C10—C9	54.1 (2)
C2—C3—C4—C5	0.3 (3)	C13—N2—C10—C9	173.61 (16)
C2—C7—C8—C1	0.5 (2)	C14—C1—C8—C7	−179.0 (2)
C2—C7—C8—C9	−178.25 (17)	C14—C1—C8—C9	−0.3 (3)
C3—C2—C7—C6	−1.2 (3)	C15—O1—C5—C4	−171.0 (2)
C3—C2—C7—C8	178.00 (18)	C15—O1—C5—C6	10.2 (3)
C3—C4—C5—O1	−179.4 (2)		

### [2-(5-Methoxy-2-methyl-1H-indol-3-yl)ethyl]trimethylazanium iodide monohydrate (umd2009b_a) Hydrogen-bond geometry (Å, º)

**Table:**

*D*—H···*A*	*D*—H	H···*A*	*D*···*A*	*D*—H···*A*
O1*W*—H1*WA*···I1^i^	0.89 (1)	2.74 (1)	3.617 (2)	168 (4)
O1*W*—H1*WB*···I1	0.89 (1)	2.76 (2)	3.618 (2)	164 (4)
N1—H1···I1^ii^	0.86 (1)	2.96 (1)	3.7416 (17)	153 (2)

Symmetry codes: (i) −*x*+1, −*y*+2, −*z*+1; (ii) −*x*+1, −*y*+1, −*z*+1.
